# Contrast-Enhanced Ultrasound in the Differential Diagnosis and Risk Stratification of ACR TI-RADS Category 4 and 5 Thyroid Nodules With Non-Hypovascular

**DOI:** 10.3389/fonc.2021.662273

**Published:** 2021-05-26

**Authors:** Yanfang Wang, Tiantian Dong, Fang Nie, Guojuan Wang, Ting Liu, Qian Niu

**Affiliations:** ^1^ Medical Center of Ultrasound, Lanzhou University Second Hospital, Lanzhou, China; ^2^ Department of Pathology, Lanzhou University Second Hospital, Lanzhou, China

**Keywords:** thyroid nodules, thyroid imaging reporting and data system, ultrasonography, contrast media, fine-needle aspiration, risk stratification

## Abstract

**Objective:**

This study aims to investigate the value of contrast-enhanced ultrasound (CEUS) in the differential diagnosis and risk stratification of ACR TI-RADS category 4 and 5 thyroid nodules with non-hypovascular.

**Methods:**

From January 2016 to December 2019 in our hospital, 217 ACR TI-RADS category 4 and 5 nodules with non-hypovascular in 210 consecutive patients were included for a derivation cohort. With surgery and/or fine-needle aspiration (FNA) as a reference, conventional ultrasound (US) features and CEUS features were analyzed. Multivariate logistic regression analysis was used to screen the independent risk factors and establish a risk predictive model. Between January 2020 and March 2021, a second cohort of 100 consecutive patients with 101 nodules were included for an external validation cohort. The model was converted into a simplified risk score and was validated in the validation cohort. The area under the receiver operating characteristic curves (AUC) were used to assess the models’ diagnostic performance.

**Results:**

Micro-calcification, irregular margin, earlier wash-out, centripetal enhancement, and absence of ring enhancement were independent risk factors and strongly discriminated malignancy in the derivation cohort (AUC = 0.921, 95% CI 0.876–0.953) and the validation cohort (0.900, 0.824–0.951). There was no significant difference (*P* = 0.3282) between the conventional US and CEUS in differentiating malignant non-hypovascular thyroid nodules, but a combination of them (the predictive model) had better performance than the single method (all *P* <0.05), with a sensitivity of 87.0%, specificity of 86.2%, and accuracy of 86.6% in the derivation cohort. The risk score based on the independent risk factors divided non-hypovascular thyroid nodules into low-suspicious (0–3 points; malignancy risk <50%) and high-suspicious (4–7 points; malignancy risk ≥ 50%), the latter with nodule ≥10mm was recommended for FNA. The risk score showed a good ability of risk stratification in the validation cohort. Comparing ACR TI-RADS in screening suitable non-hypovascular nodules for FNA, the risk score could avoid 30.8% benign nodules for FNA.

**Conclusions:**

CEUS is helpful in combination with conventional US in differentiating ACR TI-RADS category 4 and 5 nodules with non-hypovascular. The risk score in this study has the potential to improve the diagnosis and risk stratification of non-hypovascular thyroid nodules.

## Introduction

The widespread use of thyroid ultrasound (US) has increased the number of asymptomatic thyroid nodules that can be detected (1, 2). However, only 5% to 15% of thyroid nodules are malignant (3, 4). The main challenge in the evaluation of thyroid nodules is to identify those malignant nodules from benign ones. Though the conventional US is considered the preferred imaging method and valuable for the differential diagnosis of thyroid nodules, overlaps can occur between benign and malignant thyroid nodules, thus reducing the diagnostic accuracy of thyroid nodules (5). Fine-needle aspiration (FNA) is an accurate and effective method in the qualitative diagnosis of thyroid nodules preoperatively. However, at least half of all biopsied nodules are benign nodules (6), and up to 30% with indeterminate cytology results (7, 8). Therefore, overdiagnosis and overtreatment of benign thyroid nodules may have occurred. Thus, new techniques improving the diagnostic accuracy of thyroid nodules while reducing the number of unnecessary FNA are required.

Since the European Federation of Societies for Ultrasound in Medicine and Biology (EFSUMB) released the Guidelines and recommendations for the hepatic use of contrast-enhanced ultrasound (CEUS), CEUS has been used for non-hepatic applications because of its advantages in visualizing the microcirculation and the dynamic enhancing process of tumors (5, 9, 10). Previous studies have shown the good performance of CEUS in differentiating benign and malignant thyroid nodules and screening appropriate nodules for FNA, as an effective supplement technique of conventional US (11–13). And the diagnostic accuracy of thyroid nodules can be increased by combining conventional US and CEUS (14, 15).

Hypo-enhancement on CEUS is considered the most precise predictor of malignancy for thyroid nodules with high sensitivity, specificity, and accuracy of 82%, 85%, and 84%, respectively (5). While iso-/hyper-enhancement on CEUS can suggest benignity (16, 17). However, in our clinical practice, one-third of nodules with American College of Radiology’s Thyroid imaging reporting and data system (ACR TI-RADS) (18) category 4 and 5 on conventional US appear iso-/hyper-enhancement on CEUS (we defined as “non-hypovascular thyroid nodules”) that turn out to be malignant. Thus, the differential diagnosis and risk estimation for non-hypovascular thyroid nodules are challenging.

To our knowledge, prior studies paid little attention to the differential diagnosis and risk estimation of non-hypovascular thyroid nodules. In this study, based on preoperative conventional US features and CEUS features, we attempt to establish a predictive model and develop a risk score to differential diagnose and stratify non-hypovascular thyroid nodules, hoping to provide some useful information for clinical decision-making of non-hypovascular thyroid nodules.

## Materials and Methods

### Patients

This retrospective study was conducted under the Declaration of Helsinki. Ethical approval was obtained from the Ethics Committee of Lanzhou University Second Hospital. All patients signed informed consent before CEUS examination. In clinical daily work, we usually suggest nodules with indeterminate or suspicious diagnosis after conventional US as target nodules for CEUS. Consecutive patients who presented to our hospital for thyroid nodules and received both conventional US and CEUS were included in this study. The inclusion criteria were as follows: (a) ACR TI-RADS category 4 and 5 nodules with greater than or equal to 10 mm in the largest diameter; (b) nodules with complete conventional US and CEUS imaging materials; (c) nodules with iso-enhancement or hyper-enhancement on CEUS; (d) confirmed by surgery or FNA. The exclusion criteria were as follows: (a) large nodules without perinodular normal parenchyma as a reference; (b) nodules with Bethesda category II not confirmed by repeated FNA or found a change in size and ACR TI-RADS category during the at least 1 year’s follow-up. For the derivation cohort, 210 consecutive patients with 217 nodules were included between January 2016 to December 2019, a second cohort of consecutive 100 patients with 101 nodules between January 2020 to March 2021 were included for the external validation. A total of 310 patients (90 males and 220 females, mean age, 48.4 ± 12.4 years; range, 18 - 76 years) were included in this study. The diameter of 318 nodules was 17.5 ± 7.3 mm (size range 10.0 – 40.0 mm).

### Conventional Ultrasound

Two radiologists with more than 5 years’ experience in conventional US and at least 3 years’ experience in CEUS performed the ultrasound examinations. They received standardized training and had experience of 50 thyroid nodules before collecting materials in this study. Conventional US images of thyroid nodules were obtained using an iU22 scanner (Philips Medical Systems, Bothell, USA) equipped with a 5- to 12-MHz linear probe and the ACUSON Sequoia scanner (Siemens Medical Solutions, Michigan, USA) with a 4- to 10-MHz linear probe. The ACUSON Sequoia scanner was not used in the derivation cohort. The patient was placed on a bed in the supine position and the neck region was fully exposed. Conventional US static images of thyroid nodules were acquired by carefully scanning the thyroid and adjacent tissues both transversely and longitudinally. The images were stored on the instrument’s internal hard drive for further analysis.

On the conventional US, the thyroid nodules were evaluated according to the following features: size (maximum diameter), solid or almost completely solid composition (no or yes); echogenicity (hyperechoic/isoechoic or hypoechoic/very hypoechoic); shape (wider-than-tall or taller-than-wide); margin (regular-smooth/ill-defined or irregular-lobulated/irregular/extrathyroidal extension); punctate echogenic foci/micro-calcification (absence or presence); and vascularity (19) (type 1, no vascularity—defined as no power Doppler flow in the periphery or within the nodule; type 2, peripheral vascularity—defined as power Doppler flow only in the periphery of the nodule; and type 3, intranodular vascularity— defined as power Doppler flow within the nodule regardless of power Doppler flow in the periphery of the nodule).

### Contrast-Enhanced Ultrasound

Contrast-enhanced ultrasound (CEUS) examination was conducted using the same instrument as the conventional US with a 3- to 9-MHz linear probe of iU 22 or a 4- to 10-MHz linear probe of Sequoia and the same radiologists. CEUS was performed using a low mechanical index (MI = 0.06) to minimize the destruction of microbubbles and the loss of artificial signals. The plane with the maximum nodular size and an appropriate amount of surrounding parenchyma was selected in each nodule for CEUS. Patients were instructed to stop swallowing and to breathe calmly throughout the process. The contrast agent (SonoVue, Bracco, Milan, Italy) was mixed with 5 ml of saline until a homogeneously mixed suspension was obtained. Then, 1.8 to 2.0 ml of the suspension was rapidly pushed into the patient’s antecubital vein *via* a 20/22-gauge probe while their body position remained unchanged. The US machine’s timer was activated while the contrast agent was injected. Each contrast imaging acquisition lasted for at least two continuous minutes, and the process was preserved on the instrument’s internal hard drive.

**Figure 1:**
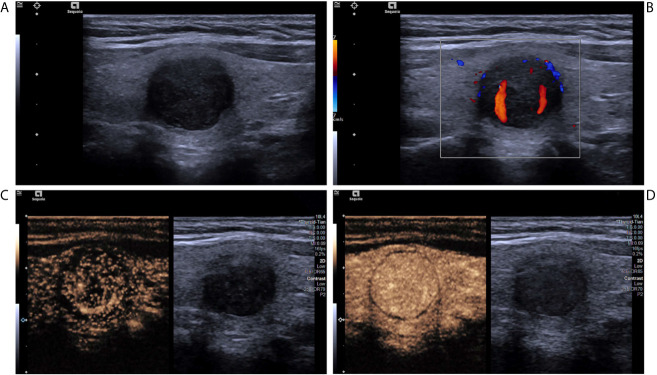
A nodular goiter with adenomatous hyperplasia in a 46-year-old woman. **(A)** Greyscale ultrasound showed that there was a solid very hypoechoic nodule in the right lobe of the thyroid, with regular margin, wider-than-tall shape, and punctate echogenic foci. The nodule was ACR TI-RADS category 5. **(B)** Color Doppler showed intranodular and peripheral vascularity. **(C)** Contrast-enhanced ultrasound revealed diffused enhancement within the nodule at the time of the 11th second after the injection of contrast agent. **(D)** Contrast-enhanced ultrasound revealed iso-enhancement at peak (the 16th second after the injection of contrast agent), with regular hypo-enhanced ring.

**Figure 2:**
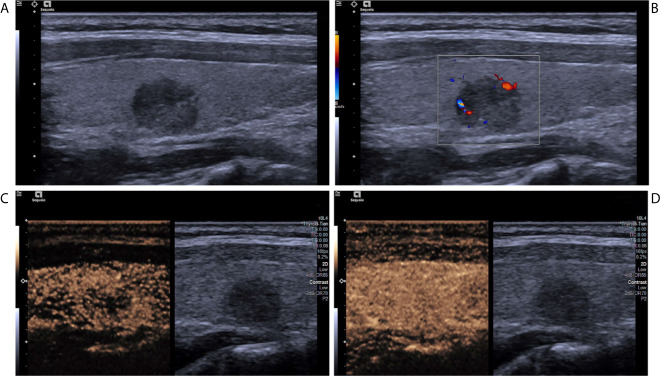
A papillary thyroid carcinoma in a 25-year-old girl. **(A)** Greyscale ultrasound showed that there was a solid hypoechoic nodule in the right lobe of the thyroid, with irregular margin, wider-than-tall shape, and punctate echogenic foci. The nodule was ACR TI-RADS category 5. **(B)** Color Doppler showed peripheral vascularity. **(C)** Contrast-enhanced ultrasound revealed the trend that contrast agent enters the nodule from the periphery to the center (centripetal enhancement) at the time of the 14th second after the injection of contrast agent. **(D)** Contrast-enhanced ultrasound revealed iso-enhancement at peak (the 22th second after the injection of contrast agent), with ill-defined enhanced border and absence of a regular enhanced ring.

**Figure 3:**
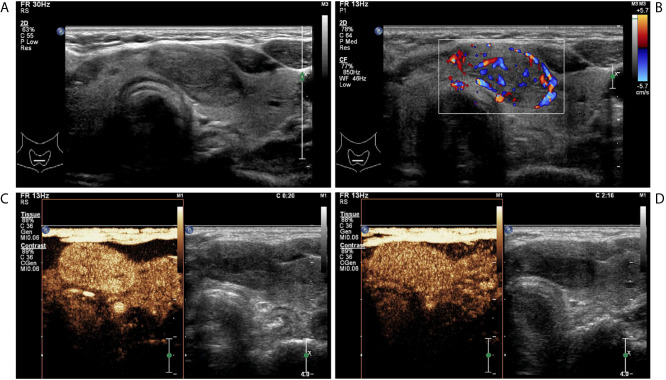
A follicular adenoma in a 63-year-old woman. **(A)** Greyscale ultrasound showed that there was a solid hypoechoic nodule in the isthmus of the thyroid, with regular margin, wider-than-tall shape, without calcification. The nodule was ACR TI-RADS category 4. **(B)** Color Doppler showed intranodular and peripheral vascularity. **(C)** Contrast-enhanced ultrasound revealed hyper-enhancement at peak (the 20th second after the injection of contrast agent), with regular hyper-enhanced ring and well-defined enhanced border. **(D)** Contrast-enhanced ultrasound revealed hyper-enhancement at the 136th second after the injection of contrast agent, which indicates later wash-out than the surrounding thyroid parenchyma.

**Figure 4:**
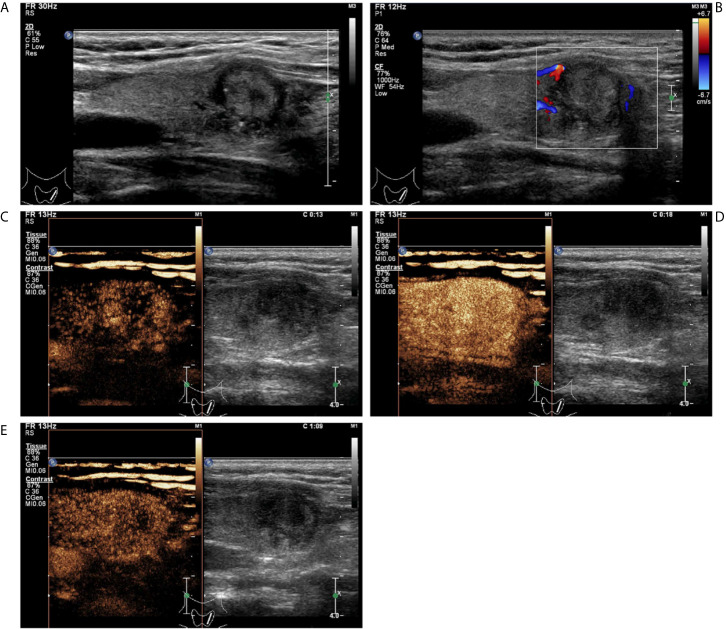
A follicular variant of papillary thyroid carcinomas in a 53-year-old woman. **(A)** Greyscale ultrasound showed that there was a solid isoechoic nodule in the left lobe of the thyroid, with irregular hypoechoic halo, irregular margin, wider-than-tall shape, and punctate echogenic foci. The nodule was ACR TI-RADS category 5. **(B)** Color Doppler showed peripheral vascularity. **(C)** Contrast-enhanced ultrasound revealed the trend that contrast agent enters the nodule from the periphery to the center (centripetal enhancement) at the time of the 13th second after the injection of contrast agent. **(D)** Contrast-enhanced ultrasound revealed heterogenous hyper-enhancement at peak (the 18th second after the injection of contrast agent), without a regular enhanced ring. **(E)** Contrast-enhanced ultrasound revealed heterogenous hypo-enhancement within the nodule at the 69th second after the injection of contrast agent, which indicates earlier wash-out than the surrounding thyroid parenchyma.

On CEUS, the thyroid nodules were evaluated for the following characteristics: homogeneity of enhancement was classified as homogeneous or heterogeneous; enhanced intensity (the perinodular normal thyroid parenchyma as a reference) was classified as iso-enhancement (**Figures 1D** and **2D**), hyper-enhancement (**Figures 3D** and **4D**), hypo-enhancement (excluded from this study), or no-enhancement (excluded from this study); ring enhancement (any regular hyper-enhanced or hypo-enhanced rim in the periphery of a nodule at the peak time) was classified as present (regular and complete) or absent (none, irregular, or incomplete); enhanced border (the boundary between the nodule and the surrounding parenchyma at the peak intensity) were classified as well-defined or ill-defined; centripetal enhancement (the contrast agent enters the nodule from the periphery of the nodule to the center) was classified as yes or no; the relative wash-in time (the time when the contrast agent entered the nodule, compared with the perinodular normal thyroid parenchyma) was classified as earlier, synchronous, or later; and the relative wash-out time (the time when the contrast agent washed out from the nodule, compared with the perinodular normal thyroid parenchyma) was classified as earlier, synchronous, or later.

Conventional US static images and CEUS cine clips were successively reviewed retrospectively by two experienced radiologists (T.T. D. and T. L.) who were blinded to the patients’ clinical information and pathology results. The two radiologists analyzed the conventional US and CEUS materials independently first and then reviewed the case with discrepancies, a consensus was reached in those cases after discussion.

### Pathological Diagnosis

In this study, all malignant nodules were confirmed by surgery, benign nodules were confirmed by surgery or FNA, and histological pathology was the final reference if both were performed. A final diagnosis of benignity was made when nodules with Bethesda category II were confirmed by repeated FNA or were found no change in size and ACR TI-RADS category during at least 1 year’s follow-up. All of the specimens were categorized by experienced pathologists (Q. N.) who were blinded to the patients’ medical history and sonographic findings.

### Model Establishment and Risk Score Construction

Stepwise logistic regression was used to select variables from 13 ultrasonographic features of non-hypovascular thyroid nodules in the derivation cohort, with pathology as the outcome. The predictive model based on significant variables will model the chance of the malignancy of thyroid nodules, with the formula given by (20–22):

$$\text{Log}\left(\frac{\pi }{1-\pi }\right)=\beta_{0}+\beta_{1}X_{1}+\beta_{2}X_{2}+\ldots \beta_{m}X_{m}$$

where π is the probability of malignancy, *β_0_* is a constant, and *β_i_* is the regression coefficient, and the *X *explains all of the significant variables.

The regression coefficient of each variable in the final model was divided by the absolute value of the smallest regression coefficient and rounded to the nearest integer. Thus, each variable was assigned an integer-weighted score. Then, the risk of malignancy of each total point was estimated. Finally, we generated a point score that divided patients into different risk classes.

### Validation of the Predictive Model and Risk Score

To validate the predictive model and the risk score, we used the data from our hospital that were not included in the derivation cohort between January 2020 and March 2021. 101 nodules in 100 consecutive patients, who met the same criteria in the derivation cohort, were included in the validation cohort.

### Statistical Analysis

SPSS software (version 22.0, IBM Corporation, Armonk, NY, USA) and MedCalc (version 15.2, Mariakerke, Belgium) was used for statistical analysis. This derivation cohort was designed to have a minimum of 195 patients so as to achieve an expected sensitivity of 0.85 and a permissible error of 0.05, at the 0.05 level of significance (two-tailed). The Shapiro-Wilk test (W test) was used to assess normal distributions of continuous variables. Normally distributed data were shown as mean ± standard deviation and analyzed with the independent samples t-test, nonnormally distributed data were shown as median and interquartile range and analyzed with the Mann-Whitney U test. Data on categorical variables were shown as numbers and percentages. The chi-squared test or Fisher’s exact test was conducted for categorical variables. Multivariate logistic regression analysis, including all of the variables from the univariate analysis that were associated with malignancy (*P*<0.1), was used in the derivation sample to ascertain the independent risk factors and establish a risk predictive model. We used the Hosmer-Lemeshow goodness-of-fit test and scatter diagram to assess model overfitting and calibration in the derivation cohort. Model’s predictive discrimination was assessed using the C statistic (area under the receiver operating characteristic curve, AUC) in the derivation cohort.

To simplify this predictive model, we created a risk scoring system, which is based on the predicted probabilities of each total point. This risk scoring system divided nodules into low-risk (malignancy rate< 50%) and high-risk (malignancy rate≥50%). To validate the model, we calculated the predicted probabilities for each nodule in the validation cohort using the predicted formula obtained in the derivation cohort. We assessed the discrimination by calculating the AUC in the validation cohort. The receiver operating characteristic (ROC) curves obtained by MedCalc were used to assess and compare the models’ predictive performance. The sensitivity, specificity, positive predictive value (PPV), negative predictive value (NPV), and accuracy were calculated at the optimal cut-off points.

## Results

### Basic Characteristics

Three hundred ten patients with 318 nodules were included in the present study. **Table 1** summarizes the demographic and clinical characteristics of the patients with non-hypovascular thyroid nodules. The derivation cohort (n = 217) and validation cohort (n = 101) did not differ in their characteristics except to the location of thyroid nodules. In the derivation cohort, 88 nodules (40.6%) were located in the upper of the thyroid lobe whereas in the validation cohort there were 23 (22.8%) located in the upper (*P* = 0.008). Patients in the derivation cohort were slightly younger, with slightly larger nodules’ size than in the validation cohort. Both cohorts showed the nodules were more common in females, without the background of Hashimoto’s thyroiditis and multifocality.

**Table 1:** Basic characteristics in the derivation and validation cohorts.

Parameters	Derivation (n = 217)	Validation (n = 101)	*P*
Gender			0.518^*^
Female	158(72.8)	70(69.3)	
Male	59(27.2)	31(30.7)	
Age (y)			
Median	48	51	0.203^#^
Interquartile	37–57	44–57	
≥55	67(30.9)	34(33.7)	0.619^*^
<55	150(69.1)	67(66.3)	
Size (mm)			
Median	16.0	15.0	0.277^#^
interquartile	12.0–21.0	11.0–21.0	
Location			0.008^*^
Upper	88(40.6)	23(22.8)	
Middle	43(19.8)	25(24.8)	
Lower	86(39.6)	53(52.5)	
Multiple			0.819^*^
No	119(54.8)	54(53.5)	
Yes	98(45.2)	47(46.5)	
Hashimoto’s thyroiditis			0.974^*^
No	178(82.0)	83(82.2)	
Yes	39(18.0)	18(17.8)	

^*^Determined with the χ^2^ test.

^#^Determined with the Mann-Whitney U test.

### Pathological Diagnosis

The derivation cohort consisted of 108 malignant nodules and 109 benign nodules. All malignant nodules (with or without FNA) were confirmed by surgery, which consisted of 74 papillary thyroid carcinomas (without subtype records), 20 classical variants of papillary thyroid carcinomas (4 with hyper-enhancement and 16 with iso-enhancement), 11 follicular variants of papillary thyroid carcinomas (5 with hyper-enhancement and 6 with iso-enhancement), one oncocytic variant papillary thyroid carcinoma with hyper-enhancement, and three follicular thyroid carcinomas (all with hyper-enhancement). While among the 109 benign nodules, 40 were determined with FNA of Bethesda category II, and 69 were confirmed by surgery, which was made up of 36 nodular goiters, 15 follicular adenomas, 11 nodular goiters with local adenomatous hyperplasia, and seven Hashimoto’s thyroiditis.

In the validation cohort, pathology demonstrated that 49 nodules (48.5%) were malignant, and 52 nodules (51.5%) were benign. All malignant nodules were confirmed by surgery, which consisted of 34 papillary thyroid carcinomas (without subtype records), 12 classical variants of papillary thyroid carcinomas, two follicular variants of papillary thyroid carcinomas, and one follicular thyroid carcinoma. Of the 52 benign nodules, 24 were determined with FNA, and 28 were confirmed by surgery, which included nine nodular goiters, five nodular goiters with adenomatous hyperplasia, 11 adenomas, and three nodular Hashimoto’s thyroiditis.

### Univariate Analysis in the Derivation Cohort

The conventional US and CEUS features and their assignments for the diagnosis of non-hypovascular benign and malignant thyroid nodules are shown in **Table 2**. There was a fair agreement for inter-observers in evaluating the ultrasonic features (kappa = 0.91). Shape, margin, and micro-calcification on the conventional US had statistical significance between the non-hypovascular benign and malignant nodules (all *P*<0.05). In benign non-hypovascular nodules, only 11 nodules (10.1%) presented micro-calcification and 93 nodules (85.3%) exhibited wider-than-tall. No significant difference was observed in the conventional US features of solid composition (*P* = 0.214), echogenicity (*P* = 0.683), and vascularity (*P* = 0.797). In comparison to benign non-hypovascular nodules, malignant non-hypovascular nodules had significant higher rate of heterogeneous enhancement (91.7% *vs*. 69.7%, *P*<0.001, **Figures 4C–E**), hyper-enhancement (63.0% *vs*. 38.6%, *P* = 0.033, **Figures 3C, D** and **4D**), absence of regular ring enhancement (86.1% *vs*. 44.0%, *P*<0.001, **Figures 2D** and **4D**), ill-defined enhanced border (91.7% *vs*. 69.7%, *P*<0.001, **Figure 2D**), centripetal enhancement (24.1% *vs*. 6.4%, *P*<0.001, **Figures 2C** and **4C**), and earlier wash-out (66.1% *vs*. 23.9%, *P*<0.001, **Figure 4E**). Synchronous wash-in were more common in the benign nodules (41.3%) than in the malignant nodules (25.9%).

**Table 2:** Sonographic features of benign and malignant non-hypovascular thyroid nodules in the derivation cohort.

Parameters	Assignment	Benign (n = 109)	Malignant (n = 108)	P^*^
Conventional ultrasound features				
Solid composition	X_1_			0.214
No	0	11(10.1)	6(5.6)	
Yes	1	98(89.9)	102(94.4)	
Echogenicity	X_2_			0.683
Hyper-/Isoechoic	0	13(11.9)	11(10.2)	
(Markedly)Hypoechoic	1	96(88.1)	97(89.8)	
Shape	X_3_			0.005
Wider-than-tall	0	93(85.3)	75(69.4)	
Taller-than-wide	1	16(14.7)	33(30.6)	
Margin	X_4_			<0.001
Regular	0	85 (78.0)	20 (18.5)	
Irregular	1	24 (22.0)	88 (81.5)	
Micro-calcification	X_5_			<0.001
No	0	98 (89.9)	44 (40.7)	
Yes	1	11 (10.1)	64 (59.3)	
Vascularity	X_6_			0.797
None	0	7 (6.4)	5 (4.6)	
Peripheral	1	35 (32.1)	33 (30.6)	
Intranodular	2	67 (61.5)	70 (64.8)	
Contrast-enhanced ultrasound features				
Homogeneity	X_7_			<0.001
Homogeneous	0	33 (30.3)	9 (8.3)	
Heterogeneous	1	76 (69.7)	99 (91.7)	
Enhanced intensity	X_8_			0.033
Iso-enhancement	0	56 (51.4)	40 (37.0)	
Hyper-enhancement	1	53 (48.6)	68 (63.0)	
Ring enhancement	X_9_			<0.001
Present	0	61 (56.0)	15 (13.9)	
Absent	1	48 (44.0)	93 (86.1)	
Enhanced border	X_10_			<0.001
Well-defined	0	93 (85.3)	45 (41.7)	
Ill-defined	1	16 (14.7)	63 (58.3)	
Centripetal enhancement	X_11_			<0.001
No	0	102 (93.6)	82 (75.9)	
Yes	1	7 (6.4)	26 (24.1)	
Wash-in	X_12_			0.002
Synchronous	0	45(41.3)	28(25.9)	
Later	1	5(4.6)	20(18.5)	
Earlier	2	59(54.1)	60(55.6)	
Wash-out	X_13_			<0.001
Synchronous	0	41(37.6)	16(14.8)	
Later	1	42(38.5)	26(24.1)	
Earlier	2	26(23.9)	66(61.1)	

*Determined with the χ^2^ test.

### Multivariate Analysis in the Derivation Cohort

**Figure 5:**
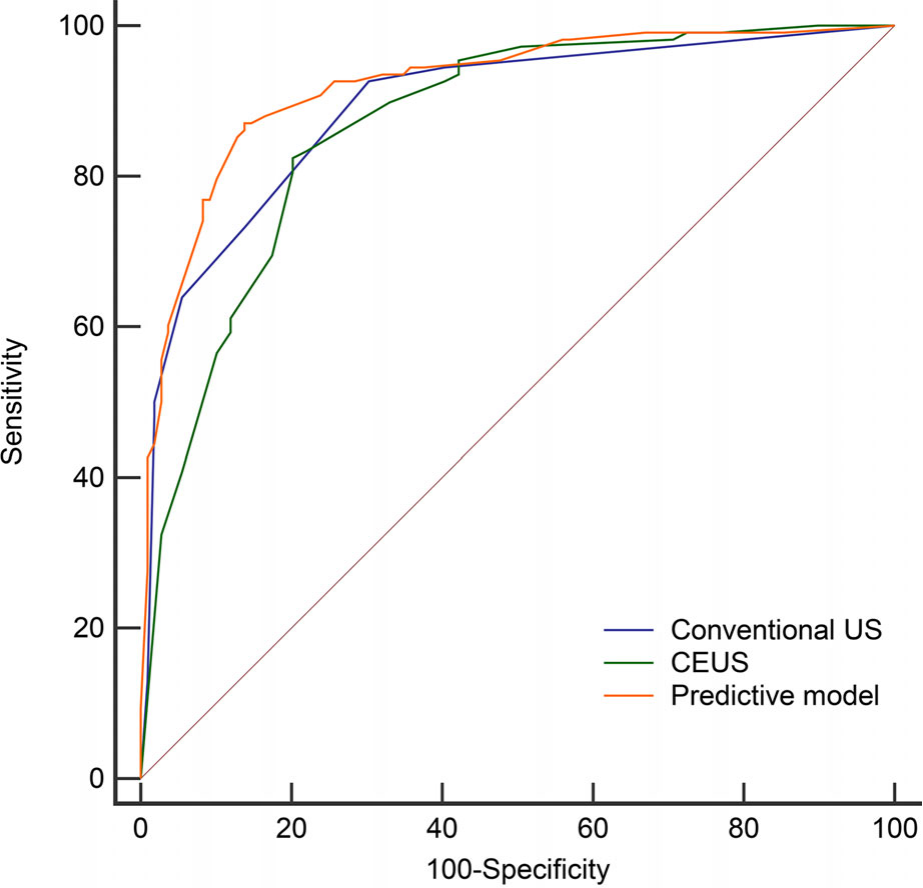
The Receiver operating characteristic curves of the conventional US (AUC=0.892), CEUS (AUC=0.868), and predictive model (AUC=0.921) in the derivation cohort.

Multivariate logistic regression identified five independent risk factors for predicting malignancy of non-hypovascular thyroid nodules: presence of micro-calcification (*X*_5_), irregular margin (*X*_4_), earlier wash-out (*X*_13_), centripetal enhancement (*X*_11_), and absence of ring enhancement (*X*_9_) (**Table 3** and **Figures 1**–**4**). Odds ratio (OR) >1 indicating risk factors and OR < 1 indicating protective factors. Micro-calcification had the largest OR value (OR = 11.9, 95% CI: 4.463–30.728), with sensitivity, specificity, and accuracy of 59.3%, 89.9%, and 74.7%, respectively. Centripetal enhancement associated with malignancy had the highest specificity (93.58%). Then a risk predictive model was established: logit (p) = −3.727 + 2.480×*X*_5_ + 2.164×*X*_4_ + 1.482×*X*_13_ + 1.254×*X*_11_ + 1.203×*X*_9_. The model showed an AUC in diagnosing non-hypovascular malignant nodules was 0.921 (95% CI, 0.876–0,953), with a sensitivity of 87.0%, specificity of 86.2%, PPV of 86.2%, NPV of 87.0%, and accuracy of 86.6%, respectively (**Table 4**, **Figure 5**), which indicated the good discriminative ability of the model to distinguish non-hypovascular nodules who were malignant from those who were benign. The P-value of the Hosmer-Lemeshow goodness-of-fit statistic with 8 degrees of freedom is 0.510. The calibration intercept and slope were 0.0369 and 0.9966 in the derivation cohort (**Supplementary Figure 1**).

**Table 3:** Multivariate analysis of the risk factors for malignancy in the derivation cohort.

Risk factors	β	*P*	OR (95%CI)	Sen (%)	Spe (%)	Acc (%)
Micro-calcification	2.480	<0.001	11.944 (4.463–30.728)	59.3	89.9	74.7
Irregular margin	2.164	<0.001	8.708 (3.570–21.240)	81.5	78.0	79.7
Earlier wash-out	1.482	0.004	4.404 (1.601–12.112)	61.1	76.2	68.7
Centripetal enhancement	1.254	0.049	3.503 (1.005–12.208)	24.1	93.6	59.0
Absence of ring enhancement	1.203	0.013	3.331 (1.293–8.578)	86.1	56.5	71.0
Constant	−3.727	<0.001	0.024	–	–	–

β, coefficient; OR, odds ration; Sen, sensitivity; Spe, specificity; Acc, accuracy.

**Table 4:** Diagnostic performance of conventional US, CEUS, and predictive model in the derivation cohort.

Methods	Sen (%)	Spe (%)	PPV (%)	NPV (%)	Acc (%)	AUC	*P*
Conventional US	92.6	69.7	95.2	90.5	81.1	0.892(0.843–0.930)	0.3282^*^
CEUS	81.5	79.8	80.2	82.1	80.6	0.868(0.815–0.910)	0.0242^#^
Predictive model	87.0	86.2	86.2	87.0	86.6	0.921(0.876–0.953)	0.0076^^^

Sen, sensitivity; Spe, specificity; PPV, positive predictive value; NPV, negative predictive value; Acc, accuracy; AUC, area under the curve.

*Conventional US vs. CEUS.

^#^Predictive model vs. Conventional US.

^^^Predictive model vs. CEUS.

Then we compared the diagnostic performance of the predictive model (a combination of conventional US and CEUS) with conventional US and CEUS alone. The AUC of the predictive model (0.921) was significantly higher than the conventional US (0.892, *P* = 0.0242) and CEUS (0.868, *P* = 0.0076) (**Table 4** and **Figure 5**). In comparison to conventional US, CEUS had a higher specificity (69.7% *vs*. 79.8%) and lower sensitivity (92.6% *vs*. 81.5%) (**Table 4**). But there was no difference (*P* = 0.3282) between the conventional US and CEUS in diagnosing non-hypovascular thyroid nodules.

### Development of Weighted Points and Establishment of the Risk Score

The weighted points of each risk factor were displayed in **Table 5**. The total points of each non-hypovascular thyroid nodule were developed using the sum of weighted points of each predictor. The malignancy risk corresponded to each total points was shown in **Table 6**. According to the observed risk of malignancy of each total points, the nodules were divided into low-suspicious (0–3 points; malignancy risk <50%) and high-suspicious (4–7 points; malignancy risk ≥ 50%) (**Table 6**). For non-hypovascular thyroid nodules (≥10 mm) with high-suspicious, FNA should be recommended.

**Table 5:** Development of weighted points for risk factors in the derivation cohort.

Risk factors	Categories	Reference value (*X*_ij_)	β_i_	β_i_ (*X*_ij_-*X*_iREF_)	Points
Micro-calcification			2.480		
	No	0 = *X*_5REF_		0	0
	Yes	1		2.480	2
Margin			2.164		
	Regular	0 = *X*_4REF_		0	0
	Irregular	1		2.164	2
Earlier wash-out			1.482		
	No	0 = *X*_13REF_		0	0
	Yes	1		1.482	1
Centripetal enhancement			1.254		
	No	0 = *X*_11REF_		0	0
	Yes	1		1.254	1
Ring enhancement			1.203		
	presence	0 = *X*_9REF_		0	0
	absence	1		1.203	1

Scores = β_i_ (X_ij_-X_iREF_)/β_8._

**Table 6:** Risk stratification according to the malignancy risk of each total points.

Risk stratification method	Total points	Risk of malignancy (%)
Low-suspicious	0	2.4
	1	7.4
	2	21.1
	3	47.1
High-suspicious	4	74.7
	5	90.8
	6	97.0
	7	99.1

### Validation of the Predictive Model and the Risk Score

**Figure 6:**
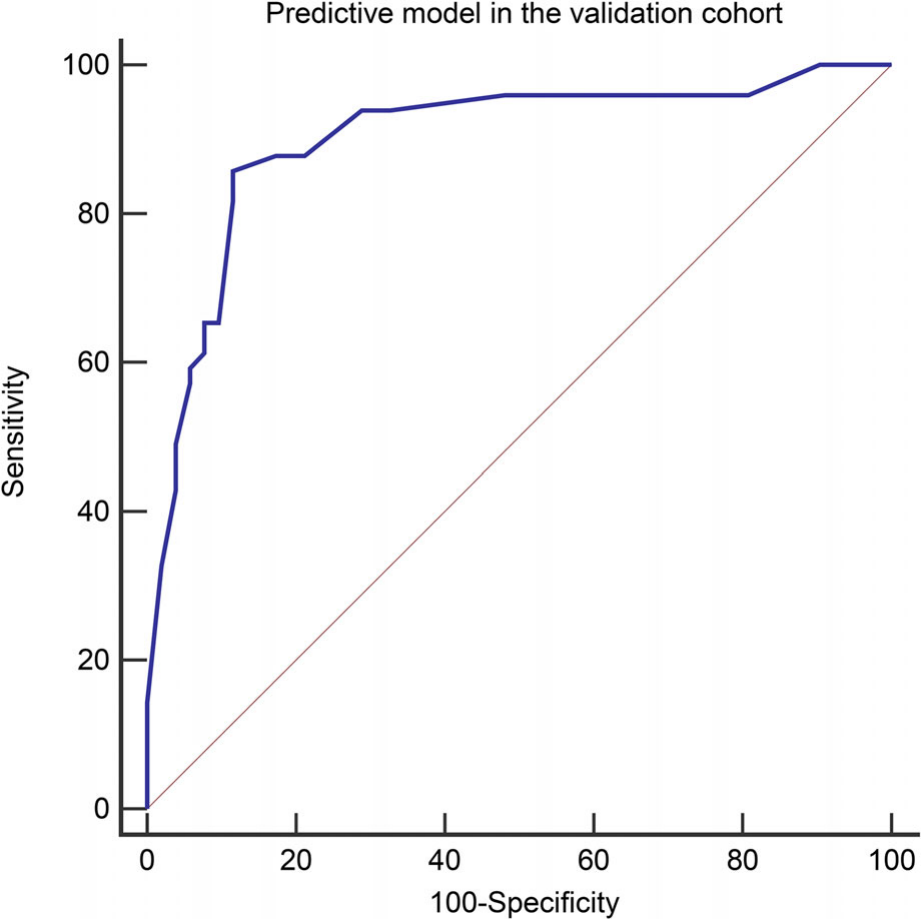
The Receiver operating characteristic curves of the predictive model in the validation cohort (AUC=0.900).

Variables used in the predictive model of the validation cohort were shown in **Table 7** (more details in **Supplementary Table 1**). The diagnostic performance of the predictive model in the validation cohort was similar to that of the derivation cohort, with an AUC, sensitivity, specificity, PPV, and NPV in the validation cohort of 0.900 (95% CI, 0.824–0.951), 85.7%, 88.5%, 87.5%, and 86.8%, respectively (**Figure 6**). The performance of the risk score in the validation cohort was summarized in **Table 8**. High accuracy (87.1%) can be observed using the risk score in the validation cohort.

**Table 7:** Features of risk factors of non-hypovascular thyroid nodules in the validation cohort.

Parameters	Assignment		Benign (n=52)	Malignant (n=49)
Conventional ultrasound features				
Margin		X_4_		
Regular		0	39(75.0)	8(16.3)
Irregular		1	13(25.0)	41(83.7)
Micro-calcification		X_5_		
No		0	41(78.8)	17(34.7)
Yes		1	11(21.2)	32(65.3)
Contrast-enhanced ultrasound features				
Ring enhancement		X_9_		
Present		0	30(57.7)	6(12.2)
Absent		1	22(42.3)	43(87.8)
Centripetal enhancement		X_11_		
No		0	47(90.4)	30(61.2)
Yes		1	5(9.6)	19(38.8)
Wash-out		X_13_		
Synchronous		0	12(25.0)	13(26.5)
Later		1	10(19.2)	6(12.2)
Earlier		2	29(55.8)	30(61.2)

**Table 8:** Validation of the risk score.

Risk score	Benign	Malignant	Sensitivity	Specificity	PPV	NPV	Accuracy
Low-suspicious	46	7	85.7%	88.5%	87.5%	86.8%	87.1%
High-suspicious	6	42					

PPV, positive predictive value; NPV, negative predictive value.

### Comparing Two Methods in Screening Non-Hypovascular Thyroid Nodules for FNA in the Derivation Cohort

According to the recommended criteria of ACR TI-RADS (18), there were 170 nodules (78.3%, 170/217) suitable for FNA. While using the risk stratification method in this study, only 92 nodules (42.4%, 92/217) were recommended for FNA (**Table 9**). Comparing ACR TI-RADS in screening suitable nodules for FNA, the risk stratification method could avoid 30.8% (69/170–9/92) benign nodules for FNA.

**Table 9:** Comparing two methods in screening non-hypovascular thyroid nodules for FNA.

	Nodules suitable for FNA	Pathology		Malignancy rate
		Benign	Malignant	
ACR TI-RADS	170	69	101	59.4%
Risk score	92	9	83	90.2%

FNA, fine-needle aspiration.

## Discussion

The early detection, accurate diagnosis of the benignity and malignancy of thyroid nodules is of considerable significance for the clinical management of thyroid nodules. Ultrasound is the preferred imaging method for diagnosing thyroid nodules. The clinical approach to thyroid nodules has recently changed to reduce the number of unnecessary biopsies while improving the diagnostic accuracy of the sonographic appearance in most detected thyroid nodules (23, 24). Therefore, improving the diagnostic accuracy of sonographic appearance while reducing the unnecessary FNA of thyroid nodules is quite crucial.

To identify the most clinically significant malignancies while reducing the number of FNA performed on benign nodules, in 2017, the ACR TI-RADS Committee presented their system for risk stratification to provide guidance regarding the management of thyroid nodules on the basis of their conventional US appearance (18). By using the ACR TI-RADS to estimate the malignancy risk of thyroid nodules, a low specificity was obtained in our medical center, especially in nodules with ACR TI-RADS category 4. This may be due to some benign nodules, such as thyroid adenoma, chronic lymphocytic thyroiditis, and even nodular goiter (for example, mummy nodule), may present as hypoechoic with an irregular margin (25). Therefore, ACR TI-RADS has some limitations in the risk stratification of thyroid nodules. Other ultrasound techniques, such as CEUS and elastography, are needed.

CEUS is considered to be an effective technique to evaluate micro-vascularization, which is much important because angiogenesis is the basis for neoplastic growth (5). Recent meta-analyses showed both the sensitivity and specificity of CEUS in diagnosing thyroid nodules were more than 82% (26, 27). Among the CEUS features of thyroid nodules, hypo-enhancement has been confirmed to be associated with malignancy by numerous studies (5, 28–32). However, not all malignant nodules appear to be hypo-enhancement on CEUS in clinical practice. Some researchers reported improved TI-RADS combined with CEUS could improve the diagnostic accuracy of thyroid nodules and reduce the number of FNA (15, 25). But they included all classifications of thyroid nodules and did not further subdivide the intensity of enhancement. In this study, we focused on ACR-TI RDS category 4 and 5 nodules only with iso-/hyper-enhancement on CEUS. The malignancy rate in the derivation cohort was only 49.8% (108/217). All of these non-hypovascular nodules were subjected to FNA and/or surgery, which may already bring the overdiagnosis and treatment. But this condition is not uncommon in many cities in China due to the patient’s anxiety and doctors’ worry about the missed diagnosis. To minimize the costs and maximize the benefits of FNA, and to prevent unnecessary diagnostic surgery (33), this study investigated the value of CEUS in the diagnosis and risk stratification of non-hypovascular thyroid nodules (≥10 mm). The results showed that CEUS is comparable to conventional US in distinguishing non-hypovascular thyroid nodules but a combination of conventional US and CEUS has superior performance than the single method. The presence of micro-calcification, irregular margin, earlier wash-out, centripetal enhancement, and absence of ring enhancement were independent risk indicators. By weighting these risk indicators, we developed a simple risk score and divided the non-hypovascular nodules into two categories: low-suspicious (0–3 points) and high-suspicious (4–7 points). By using the risk score, 30.8% of benign nodules could avoid FNA.

The well-accepted conventional US features associated with malignancy include solid composition, (markedly) hypoechoic, taller-than-wide, irregular margin, and microcalcification (18, 34). In the present study, we not only analyzed the above features but also the vascularity by color Doppler, as we focused on the non-hypovascular thyroid nodules. The results showed that micro-calcification and irregular margin were correlated with malignancy for non-hypovascular thyroid nodules, with sensitivity and specificity of 59.3% and 89.9%, 81.5% and 78.0%, respectively. This indicated no single feature was sensitive and specific enough in the diagnosis of non-hypovascular thyroid nodules. Although a taller-than-wide shape was more frequently found in malignant nodules, it was not specific for non-hypovascular nodules. Solid composition, (markedly) hypoechoic, and vascularity were not independent risk factors in the current study. This can be explained by the fact that 92.2% (200/217) of the non-hypovascular thyroid nodules were solid composition and 88.9% (193/217) with (markedly) hypo-echogenicity in the derivation cohort. The vascularity assessed by Doppler ultrasound in the diagnosis of thyroid nodules is controversial. Some authors claim that intranodular vascularity is associated with malignancy for thyroid nodules (35, 36), while others demonstrate that it is not helpful to predict malignancy (19, 37). In our study, the results showed the vascularity was less helpful in differentiating non-hypovascular thyroid nodules. This may be to the low sensitivity of color Doppler in detecting vascularity within thyroid nodules.

CEUS can make up for the low sensitivity of color Doppler in detecting vascularity within tumors. Though CEUS can be analyzed qualitatively or quantitatively, there is no unified CEUS terminology for qualitative or quantitative analysis of thyroid nodules at present. And no single CEUS features seem to be sufficiently sensitive or specific to distinguish between benign and malignant thyroid nodules. A previous meta-analysis found that qualitative evaluation acquired better sensitivity and specificity than quantitative evaluation in differentiating thyroid nodules (38). In this study, we analyzed seven qualitative CEUS variables (homogeneity, enhanced intensity, ring enhancement, enhanced border, centripetal enhancement, relative wash-in time, and relative wash-out time). The results of the univariate analysis in the derivation cohort showed that all variables were significantly different between benign and malignant non-hypovascular nodules. We observed that non-hypovascular malignant thyroid nodules on CEUS could be heterogeneous, hyper-enhancement, absence of ring enhancement, ill-defined enhanced border, centripetal enhancement, earlier wash-in, and earlier wash-out. These findings seemed to be not exactly the same as Wu et. al’s (39), which concluded later arrival, centripetal mode of entrance, hypo-enhancement, heterogeneous enhancement, and earlier wash-out were CEUS diagnostic criteria of malignant nodules. This may be due to the select difference as we exclude nodules with hypo-enhancement and non-enhancement. Different enhancement modes are related to the corresponding pathologic mechanisms (40). Numerous studies (11, 32, 41–44) have demonstrated hypo-enhancement and heterogeneous enhancement are major features of malignancy thyroid nodules. Possible explanations were necrosis, calcification, fibrosis, and embolus formation within the tumor (43). The reason for those malignant nodules appeared iso-/hyper-enhancement on CEUS is unclear yet. In the derivation cohort, we found 3(100%) follicular thyroid carcinomas, 5 (45.4%, 5/11) follicular variants of papillary thyroid carcinomas, and 4 (20%, 4/20) classical variants of papillary thyroid carcinomas all with hyper-enhancement. As follicular tumors are usually hyper-vascular, it is possible to speculate that non-hypovascular malignant nodules with hyper-enhancement on CEUS may be more correlated with follicular thyroid carcinoma and follicular variants of papillary thyroid carcinoma. Unfortunately, there was no pathological record on the variants of 74 papillary thyroid carcinoma in this study. We will further study them shortly.

In general, tumor neovascularization is relatively dense in the marginal zone and sparse in the center, which may lead to centripetal enhancement and heterogeneous enhancement in the malignant nodules (39). In addition, the immature new blood vessels usually have a low resistance index and may exist arteriovenous fistula, both earlier wash-in and earlier wash-out might be present during the CEUS process. A thin and regular ring enhancement is a feature of benign nodules, especially for follicular adenoma. When the malignant nodules grow unevenly and invade the surrounding normal parenchyma, the ring may be incomplete or blurred, thus may resulting in absence of ring enhancement and ill-defined enhanced border. In this study, the results showed that earlier wash-out, centripetal enhancement, and absence of ring enhancement were independent CEUS indicators associated with malignancy for non-hypovascular nodules. Centripetal enhancement had the highest specificity (93.6%) and the absence of ring enhancement had the highest sensitivity (86.1%) in diagnosing non-hypovascular malignant thyroid nodules, which could attribute to an increase in accuracy of distinguishing benign nodules and prevent patients with a further invasive procedure. Similar results were also reported by other researchers (11, 30, 45).

A prior study reported the diagnostic accuracy of contrast-enhanced ultrasound (CEUS) for distinguishing malignant thyroid nodules from benign thyroid nodules remains controversial (26). In our study, CEUS is comparable to conventional US in distinguishing non-hypovascular thyroid nodules. And CEUS had high specificity than conventional US (79.8% *vs*. 69.7%) in differential diagnosing non-hypovascular nodules, which indicated a better capability of identifying benign nodules and avoiding unessential FNA. By a combination of conventional US and CEUS, Ma et al. (46) reported that the sensitivity can up to 89.4% (84/94) and specificity of 93.6% (73/78). Xu et al. (47) demonstrated that the sensitivity, specificity, and AUC of the combined method were 85.7%, 83.3%, and 0.867, respectively. Our study also confirmed the good diagnostic efficacy of a combination for diagnosing non-hypovascular thyroid nodules, with an AUC, sensitivity, and specificity of 0.921, 87.0%, and 86.2% in the derivation cohort and 0.900, 85.7%, and 88.5% in the external validation cohort, respectively. The AUC of the combined method in the derivation cohort in this study was significantly higher than the conventional US (*Z* = 2.255, *P* = 0.0242) and CEUS (*Z* = 2.671, *P* = 0.0076) alone (**Figure 5** **)**, which was in line with published articles (14, 15).

Recently, some studies have also reported prediction models for differentiating benign and malignant thyroid nodules (22, 42, 48–50). However, few of them weighted for the risk factors and it was inconvenient to calculate the risk of malignancy clinically. In this study, we weighed for these risk factors and developed a risk score, which could divide the non-hypovascular nodules into different risk classes with a simpler method. By using this risk score in the validation cohort, the risk score exhibited the good ability of risk stratification, with a sensitivity of 85.7%, sensitivity of 88.5%, and accuracy of 87.1%, respectively. Moreover, by comparing ACR TI-RADS and risk score in screening suitable nodules for FNA, we found the risk score could avoid 30.8% benign nodules for FNA. This meant the risk score had better performance than ACR TI-RADS in the clinical management of non-hypovascular thyroid nodules. The reduced number of FNA mainly comes from the ACR TI-RADS category 4. Therefore, the risk score can help us distinguish non-hypovascular thyroid nodules (especially for ACR TI-RADS category 4) more simply, avoid unnecessary FNA, and improve the malignancy rate of FNA.

At present, few reports describe the risk stratification of non-hypovascular thyroid nodules using conventional US features and CEUS features. This study provides a simple and practical risk score to estimate the malignancy risk level of non-hypovascular thyroid nodules. Using this risk score, radiologists could diagnose and stratify the non-hypovascular thyroid nodules more conveniently and accurately, helping screening necessary nodules for further FNA. However, this study has several limitations. First, a selection bias may be present that we excluded nodules with no-enhancement and hypo-enhancement. Second, the malignant thyroid nodules were mainly papillary thyroid carcinoma, while other histological types were rare and the variants of 74 papillary thyroid carcinoma were unclear. Third, as the present study was a single-center study with small sample size, additional multi-center studies, and larger sample sizes are needed in the future.

## Conclusions

In conclusion, we developed a risk score based on significant conventional US features and CEUS features to differential diagnose and stratify non-hypovascular thyroid nodules. The risk score was validated externally and prove to be reproducible with good performance. CEUS is comparable to conventional US in distinguishing non-hypovascular thyroid nodules, but a combination of them has the potential to improve the individualized management of non-hypovascular thyroid nodules, avoiding unnecessary FNA for benign nodules.

## Data Availability Statement

The original contributions presented in the study are included in the article/**Supplementary Material**. Further inquiries can be directed to the corresponding author.

## Ethics Statement

The studies involving human participants were reviewed and approved by Ethics Committee of Lanzhou University Second Hospital. The patients/participants provided their written informed consent to participate in this study.

## Author Contributions

Conception and design of the study: YW and FN. Ultrasound data acquisition: YW and GW. Clinical and pathological data collection: GW and QN. Analysis and interpretation of data: TD and TL. Drafting the manuscript: YW. Revising and final approval of the version to be published: TD and FN. All authors contributed to the article and approved the submitted version.

## Funding

This research was supported by the Key Talent Projects of Gansu Province in 2019 (Grant number: 2019RCXM021).

## Conflict of Interest

The authors declare that the research was conducted in the absence of any commercial or financial relationships that could be construed as a potential conflict of interest.
