# Supplementary material for: Contrast-Enhanced Ultrasound in the Differential Diagnosis and Risk Stratification of ACR TI-RADS Category 4 and 5 Thyroid Nodules With Non-Hypovascular
Source: Front Oncol. 2021 May 26;11:662273. doi: 10.3389/fonc.2021.662273 (PMC8189148; doi:10.3389/fonc.2021.662273)
Supplement: Supplementary file 1 [file DataSheet_1.pdf]

Supplementary Tables

Table 1 Sonographic features of benign and malignant non-hypovascular thyroid nodules in the validation cohort

Parameters	Assignment	Benign(n=52)	Malignant(n=49)
Conventional ultrasound features			
Solid composition	X_1		
No	0	5(9.6)	2(4.1)
Yes	1	47(90.4)	47(95.9)
Echogenicity	X_2		
Hyper-/Isoechoic	0	12(23.1)	2(4.1)
(Markedly)Hypoechoic	1	40(76.9)	47(95.9)
Shape	X_3		
Wider-than-tall	0	43(82.7)	35(71.4)
Taller-than-wide	1	9(17.3)	14(28.6)
Margin	X_4		
Regular	0	39(75.0)	8(16.3)
Irregular	1	13(25.0)	41(83.7)
Micro-calcification	X_5		
No	0	41(78.8)	17(34.7)
Yes	1	11(21.2)	32(65.3)
Vascularity	X_6		
None	0	5(9.6)	1(2.0)
Peripheral	1	20(38.5)	14(28.6)
Intranodular	2	27(51.9)	34(69.4)
Contrast-enhanced ultrasound features			
Homogeneity	X_7		
Homogeneous	0	18(34.6)	14(28.6)
Heterogeneous	1	34 (65.4)	35(71.4)
Enhanced intensity	X_8		
Iso-enhancement	0	22(42.3)	30(61.2)
Hyper-enhancement	1	30(57.7)	19(38.8)
Ring enhancement	X_9		
Present	0	30(57.7)	6(12.2)
Absent	1	22(42.3)	43(87.8)
Enhanced border	X_{10}		
Well-defined	0	40(76.9)	17(34.7)
Ill-defined	1	12(23.1)	32(65.3)
Centripetal enhancement	X_{11}		
No	0	47(90.4)	30(61.2)
Yes	1	5(9.6)	19(38.8)
Wash-in	X_{12}		
Synchronous	0	23(44.2)	16(32.7)
Later	1	1(1.9)	11(22.4)
Earlier	2	28(53.8)	22(44.9)
Wash-out	X_{13}		
Synchronous	0	12(25.0)	13(26.5)
Later	1	10(19.2)	6(12.2)
Earlier	2	29(55.8)	30(61.2)

Supplementary Figure

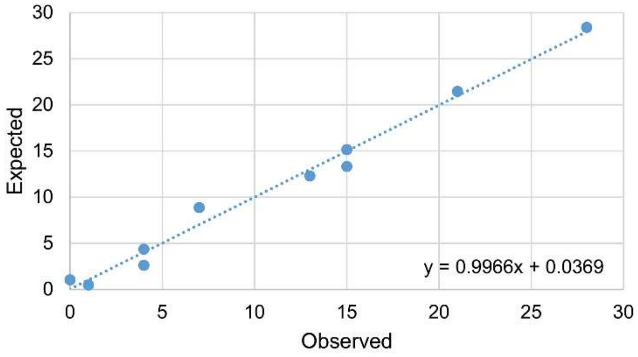

Supplementary Figure 1 The calibration curve of the predictive model in derivation cohort.
